# Supplementary material for: Deciphering spatial domains from spatial multi-omics with SpatialGlue
Source: Nat Methods. 2024 Jun 21;21(9):1658–67. doi: 10.1038/s41592-024-02316-4 (PMC11399094; doi:10.1038/s41592-024-02316-4)
Supplement: Supplementary file 1 — Supplementary Notes, Methods, Tables 1–5 and Figs. 1–15 [file 41592_2024_2316_MOESM1_ESM.pdf]

nature methods

Supplementary information https://doi.org/10.1038/s41592-024-02316-4

Deciphering spatial domains from spatial multi-omics with SpatialGlue

In the format provided by the authors and unedited

Supplementary information for 'Deciphering spatial domains from spatial multi-omics with SpatialGlue'

Simulation data generation

To provide data samples with ground truth for evaluation, we followed the approach outlined by Townes et al. [1] to use nonnegative spatial factorization to simulate spatial multi-omics data. We employed the 'ggblocks' model from Townes et al. to generate expression matrices of different modalities. For modality 1, we generated a spatial gene expression matrix of 1,296 cells x 1,000 genes with the zero-inflated negative binomial (ZINB) distribution, featuring 4 distinct factors. Similarly, we generated a spatial protein expression matrix (modality 2) with negative binomial (NB) distribution, dimensions of 1,296 cells x 100 proteins, and featuring 4 distinct factors. The simulation approach recapitulates the ZINB and NB distributions of spatial transcriptomics and proteomics respectively and matches the cells from the two modalities. We also added Gaussian distributed noise to both modalities to better mimic real-world scenarios. To increase statistical analysis power, we generated 5 simulation datasets with different parameters. The summary statistics of the 5 simulation datasets are shown in Table S3.

Benchmarking methods

To evaluate the performance of SpatialGlue, we compare it with 10 state-of-the-art methods, including 7 single-cell multi-omics data integration methods, Seurat [2], totalVI [3], MultiVI [4], MOFA+ [5], MEFISTO [6], scMM [7], and StabMap [8], and 3 single-modal methods, SpaGCN [9], STAGATE [10], and GraphST [11].

All of benchmarking methods were executed based on their provided vignettes. For Seurat's data pre-processing of the RNA modality, 2,000 and 3,000 highly variable genes were selected for log normalization for the RNA & protein and RNA & ATAC (histone) data, respectively. The dimensions of feature reduction were set to 30 and 18 for the RNA and protein modalities, respectively. For the RNA & ATAC (histone) data, the dimensions of feature reduction were set to 10 for both the RNA and ATAC (histone) modalities. For MOFA+ and MEFISTO, the top 2,000 and 5,000 highly variable genes and peaks were chosen for normalization for the RNA and ATAC (histone) modality data, respectively. The number of factors was set to 10. For totalVI and MultiVI, we employed the scVI package (version 1.0.2) for data integration. The input dataset was preprocessed using the standard SCANPY workflow. Specifically, the top 4,000 highly variable genes were selected for log-normalization when implementing totalVI tool. For MultiVI, genes and peaks expressed in fewer than 1% pixels were eliminated for the RNA & epigenome (ATAC, histone) data. We need to highlight that totalVI was designed only for CITE-seq. scMM was executed with its default settings. The epochs, batch size, and learning rating were set to 50, 32, and 0.0001, respectively. Following the tutorial provided by the original paper, we ran StabMap using default settings. SpaGCN, STAGATE, and GraphST are deep learning models designed for single-modal spatial transcriptomics data. All three models were employed based on the tutorials provided. To adapt these methods to spatial multi-omics data, we concatenated the pre-processed expression matrices of the RNA and protein/ATAC/histone data as input to obtain latent representations. For totalVI, MultiVI, scMM, SpaGCN, STAGATE, and GraphST, after model training, we extracted the latent representations to perform clustering with the 'mclust' algorithm [12].

Downstream analyses

Spatial clustering. Taking the expression data of different omics modalities as input, SpatialGlue outputs an integrated representation of spots/cells. With the output representations as input, we applied the 'mclust' algorithm [12] to identify spatial domains. We tested different numbers of clusters to select the clustering that best capture the known biological structures and/or cell types.

DEG analysis. After obtaining the clustering labels, differential expressed gene (DEG) analysis was performed on the identified clusters using Seurat v4.0 [2] to identify differentially expressed genes, proteins, or peaks. Similarly, tSNE and UMAP plots were generated using the integrated representations for visualization.

Signac. We first performed log-normalization followed by data scaling on the 'SCT' assay using the Seurat package. To find differentially expressed genes, we used the 'FindAllMarkers' function with the following parameter settings: logfc.threshold = 0.1, min.pct = 0.1 and 'wilcoxon' test. We then ran Term frequency-inverse document frequency (TF-IDF) normalization, FeatureSelection and RunSVD (Singular value decomposition), followed by data scaling on the CUT&Tag assay using Signac v1.8.0 [13]. We estimated the Gene Activity scores and used the SpatialGlue's clustering results to identify differentially expressed genes using the Gene Activity Scores using the 'FindAllMarkers' function with the following parameters settings: logfc.threshold = 0.25, min.pct = 0.25 and 'wilcoxon' test.

ArchR. To estimate the differentially expressed peaks, we employ the ArchR package v1.0.2 [14]. We first created 'arrow' files using the parameters: minFrags = 0, maxFrags = 1e+07, tile_size = 5000, and 'the mm10' genome. We computed the dimensionality reduced space via IterativeLSI with dims = 1:30 and performed clustering using the standard Seurat neighborhood detection method via addClusters, followed by UMAP via the 'addUMAP' function. We then prepare the spatially resolved ATAC object as follows: the spatial information was integrated using Seurat's 'Read10X_image' function to create a 10x Genomics Visium object named image containing all the spatial folder information. We then filtered out the off-tissue pixels in both the image object and in the ArchR object. The gene score matrix containing all gene accessibility scores and metadata including the computed SpatialGlue clusters was then extracted, and the image object is added.

We next generated a reproducible peak set in ArchR using the 'addReproduciblePeakSet' function and called the peaks using MACS2 [15]. The differentially expressed peaks were then identified for the SpatialGlue's clusters in the 'PeakMatrix' with the 'getMarkerFeatures' function. Marker genes with differential gene scores were also computed from the 'GeneScoreMatrix' using the same function. Finally, we computed the linkage between genes and peaks using the 'addPeak2GeneLinks' function with the 'Iterative LSI' reductions and 'GeneScoreMatrix' values. We ran the 'addPeak2GeneLinks' function with the following settings: corCutOff = 0.45 and resolution = 1,000. To visualize the correlation between peaks and genes, we used the 'plotPeak2GeneHeatmap' function to plot the peak-to-gene links heatmap.

Evaluation metrics

To evaluate the data integration performance of the model, we used eight quantitative metrics, of which six are supervised metrics (AMI, NMI, ARI, homogeneity, mutual information, and V-measure), and two unsupervised metrics (Jaccard similarity and Moran's *I* score). The supervised metrics were computed using the scikit-learn [16] package in Python.

ARI. ARI (Adjusted Rand Index) measures the similarity between the ground truth and predicted labels. If C is a set of ground truth assignments and K the set of predicted labels, let us define a and b as:

- a, the number of pairs of elements that are in the same set in C and in the same set in K.
- b, the number of pairs of elements that are in different sets in C and in different sets in K.

The unadjusted Rand index is then given by:

$$RI = \frac{a+b}{C_2^{n_{samples}}}, \quad (1)$$

where $C_2^{n_{samples}}$ is the total number of possible pairs in the dataset. However, the Rand Index (RI) does not guarantee that random label assignments will get a value close to zero, especially if the number of clusters is in the same order of magnitude as the number of samples. To counter this effect, ARI is introduced to discount the expected RI $E[RI]$ of random labellings, and is defined as follows:

$$ARI = \frac{RI - E[RI]}{\max(RI) - E[RI]}. \quad (2)$$

Mutual Information. MI (Mutual Information), NMI (Normalized Mutual Information), and AMI (Adjusted Mutual Information) are functions that measure the agreement of the ground truth and predicted labels. Assume two label assignments (of the same N objects), U and V. Their entropy is the amount of uncertainty for a partition set, defined by:

$$H(U) = -\sum_{i=1}^{|U|} P(i)\log(P(i)), \quad (3)$$

where $P(i) = \frac{|U_i|}{N}$ is the probability that an object picked at random from U falls into class U_i. Likewise for V:

$$H(V) = -\sum_{j=1}^{|V|} P'(j)\log(P'(j)), \quad (4)$$

with $P'(j) = \frac{|V_i|}{N}$. The mutual information (MI) between U and V is calculated by:

$$MI(U,V) = \sum_{i=1}^{|U|}\sum_{j=1}^{V} P(i,j)\log\left(\frac{P(i,j)}{P(i)P'(j)}\right), \quad (5)$$

where $P(i,j) = \frac{|U_i \cap V_j|}{N}$ is the probability that an object picked at random falls into both classes U_i and V_j. The normalized mutual information is defined as

$$NMI(U,V) = \frac{MI(U,V)}{mean(H(U),H(V))}. \quad (6)$$

The AMI can be defined as follows:

$$AMI = \frac{MI - E[MI]}{mean(H(U),H(V) - E[MI])}, \quad (7)$$

where $E(MI)$ means the expected value of the mutual information and is defined as follows:

$$E[MI(U,V)] = \sum_{i=1}^{|U|}\sum_{j=1}^{|V|}\sum_{n_{ij}=(a_i+b_j-N)^+}^{\min(a_i,b_j)} \frac{n_{ij}}{N}\log\left(\frac{N.n_{ij}}{a_i b_j}\right)\frac{a_i!\,b_j!\,(N-a_i)!\,(N-b_j)!}{N!\,n_{ij}!\,(a_i-n_{ij})!\,(b_j-n_{ij})!\,(N-a_i-b_j+n_{ij})!},$$

(8)

where $a_i = |U_i|$ and $b_j = |V_j|$ are the numbers of elements in U_i and V_j, respectively.

Homogeneity Homogeneity is a clustering metric that measures if each cluster contains only members of a single class. This metric is grounded in the concept that a cluster should be composed entirely of data points that are similar to each other. The value of homogeneity ranges from 0 to 1, where 1 signifies perfect homogeneity and 0 indicates that the clustering is completely heterogeneous.

V-measure V-measure is a metric that combines both homogeneity and completeness to provide a single score that measures the quality of a clustering outcome. Homogeneity measures if each cluster contains only members of a single class, while completeness evaluates whether all members of a given class are assigned to the same cluster. V-measure is thus a harmonic mean of homogeneity and completeness, providing a balance between these two aspects. The homogeneity and completeness scores are formally given by:

$$h = 1 - \frac{H(C|K)}{H(C)}, \quad (9)$$

$$c = 1 - \frac{H(K|C)}{H(K)}, \quad (10)$$

where $H(C|K)$ is the conditional entropy of the classes given the cluster assignments and is given by:

$$H(C|K) = -\sum_{c=1}^{|C|}\sum_{k=1}^{|K|}\frac{n_{c,k}}{n}\cdot\log\left(\frac{n_{c,k}}{n_k}\right), \quad (11)$$

and $H(C)$ is the entropy of the classes and is given by:

$$H(C) = -\sum_{c=1}^{|C|}\frac{n_c}{n}\cdot\log\left(\frac{n_c}{n}\right), \quad (12)$$

with n the total number of samples, n_c and n_k the number of samples respectively belonging to class c and cluster k, and finally $n_{c,k}$ the number of samples from class c assigned to cluster k. The conditional entropy of clusters given class $H(K|C)$and the entropy of clusters $H(K)$ are defined in a symmetric manner. Finally, V-measure is defined as the harmonic mean of homogeneity and completeness:

$$v = 2 * \frac{h*c}{h+c}. \quad (13)$$

Both homogeneity score and V-measure score range from 0 to 1, where 1 indicates perfect clustering and 0 imply poor clustering.

Jaccard similarity. Similar to the metric employed by Ghazanfar et al. [8], for spot i, we separately extract the sets N_{im} and N_{ie} of size k (default 50) containing the nearest spots in the m-th modality and embedding space, that is,

$$N_{im} = \left\{set\ of\ neighbors\ of\ feature\ space\ m,\ s.t.\ rank\left(D\left(Z_{im}, Z_{jm}\right)\right) \le k\right\}, \quad (14)$$

where $D(a,b)$ is the Euclidean distance of vectors a and b. The Jaccard similarity is thus:

$$J_i = Jaccard(N_{im}, N_{ie}) = \frac{|N_{im} \cap N_{ie}|}{|N_{im} \cup N_{ie}|}. \quad (15)$$

A larger value of J_i means greater similarity between the integrated representation and the m-th modality data.

Moran's I score. Moran's I score was calculated using the Squidpy package (Palla et al.[17]). Briefly, given a feature (gene or label) and the spatial location of observations, Moran's I score assesses whether the pattern expressed is clustered, dispersed, or random (Getis et al.[18]). Specifically, Moran's I is defined as:

$$I = \frac{n}{W} \frac{\sum_i^n \sum_j^n w_{i,j} z_i z_j}{\sum_{i=1}^n z_i^2}, \quad (16)$$

where z_i is the deviation of the feature from the mean $(x_i - \tilde{X})$, $w_{i,j}$ is the spatial weight between observations, n is the number of spatial units, and W is the sum of all $w_{i,j}$. A higher value of Moran's *I* score corresponds to a more centralized spatial pattern.

Neighborhood enrichment and co-occurrence. To assess the spatial relationships between clusters, we calculated neighborhood enrichment and co-occurrence scores with the Squidpy [17] package. First, we applied the 'mclust' algorithm on the output representation of SpatialGlue to obtain spatial clusters. With the spatial clusters as input, we calculated the co-occurrence score of each cluster using Squidpy. For neighborhood enrichment analysis to evaluate spatial autocorrelations of clusters, we used the spatial clusters and coordinates as input to Squidpy.

Ablation studies

Here we performed a series of ablation studies to illustrate the impact of different components in the SpatialGlue model on performance. We first considered the use of attention (A) over concatenation (C) in integrating information. We created three variants of SpatialGlue (AC, CA, CC). The variants CA and CC clearly showed deterioration in capturing the original data and AC was the closest in performance to the original SpatialGlue (Suppl. Figure S1c). This was also reflected in the computed supervised metrics (Suppl. Figure S1d). We next demonstrated the importance of spatial information by feeding in data without spatial information. Without spatial information, the output of the variant was much noisier than that of SpatialGlue (Suppl. Figure S1e). The metrics also showed that this variant performed worse (Suppl. Figure S1f). Finally, we fed the original data into SpatialGlue and found no performance difference (Suppl. Figure S1g,h). This suggested that PCA pre-processing does not negatively impact performance while offering the benefit of reduced data dimension which lower memory requirements and speed up computation.

Finally, we also tested SpatialGlue alongside single-modal methods with simple data concatenation of simulated and experimentally acquired data. With simulated data, GraphST and SpatialGlue achieved similar performance while STAGATE and SpaGCN's outputs were noisier (Suppl. Figure S5b,c). The second test employed the P22 mouse brain data with RNA-Seq and ATAC-Seq modalities (Suppl. Figure S5d,c,e). STAGATE's output showed high levels of smoothing (Moran's I score) but lowest similarity to the data modalities (lowest Jaccard Similarity). SpaGCN achieved the lowest Moran's I score and also failed to delineate the cortex layers. GraphST and SpatialGlue obtained similar Moran's I score but SpatialGlue was the overall best in terms of Jaccard Similarity. Visually, SpatialGlue was also able to capture the cortex layers more accurately.

Sensitivity to parameters

Using the simulated data, we tested SpatialGlue's sensitivity to parameter changes, namely the number of neighbors *k*, the number of PCs, and the number of GNN layers. The performance of SpatialGlue clearly varied significantly with *k* increasing both visually and in terms of metrics (Extended Data Fig.1a,b). At relatively small values of *k* (3 to 6), we considered the performance loss to be tolerable. By default, we set *k* to 3. For number of PCs selected, the performance increased with the PCs count increasing but reduced from 25 to 50. The initial increase could be attributed to the additional PCs capturing more information while the poor performance at 50 was likely due to the higher dimension PCs containing noise instead. We also evaluated SpatialGlue's performance with the number of GNN layers ranging from 1 to 3. Our results showed SpatialGlue achieving the best performance with 1 GNN layer. Therefore, we use this value as the default in our model.

Reference

1. Townes, F. W. & Engelhardt, B. E. Nonnegative spatial factorization applied to spatial genomics. *Nat. Methods* **20**, 229–238 (2023).
2. Hao, Y. *et al.* Integrated analysis of multimodal single-cell data. *Cell* **184**, 3573-3587.e29 (2021).
3. Gayoso, A. *et al.* Joint probabilistic modeling of single-cell multi-omic data with totalVI. *Nat. Methods* **18**, 272–282 (2021).
4. Ashuach, T. *et al.* MultiVI: deep generative model for the integration of multimodal data. *Nat. Methods* **20**, 1222–1231 (2023).
5. Argelaguet, R. *et al.* MOFA+: A statistical framework for comprehensive integration of multi-modal single-cell data. *Genome Biol.* (2020) doi:10.1186/s13059-020-02015-1.
6. Velten, B. *et al.* Identifying temporal and spatial patterns of variation from multimodal data using MEFISTO. *Nat. Methods* **19**, 179–186 (2022).
7. Minoura, K., Abe, K., Nam, H., Nishikawa, H. & Shimamura, T. A mixture-of-experts deep generative model for integrated analysis of single-cell multiomics data. *Cell reports methods* **1**, 100071 (2021).
8. Ghazanfar, S., Guibentif, C. & Marioni, J. C. Stabilized mosaic single-cell data integration using unshared features. *Nat. Biotechnol.* (2023) doi:10.1038/s41587-023-01766-z.
9. Hu, J. *et al.* SpaGCN: Integrating gene expression, spatial location and histology to identify spatial domains and spatially variable genes by graph convolutional network. *Nat. Methods* **18**, 1342–1351 (2021).
10. Dong, K. & Zhang, S. Deciphering spatial domains from spatially resolved transcriptomics with an adaptive graph attention auto-encoder. *Nat. Commun.* **13**, 1739 (2022).
11. Long, Y. *et al.* Spatially informed clustering, integration, and deconvolution of spatial transcriptomics with GraphST. *Nat. Commun.* **14**, 1155 (2023).
12. Scrucca, L., Fop, M., Murphy, T. B. & Raftery, A. E. mclust 5: Clustering, Classification and Density Estimation Using Gaussian Finite Mixture Models. *R J.* **8**, 289–317 (2016).
13. Stuart, T., Srivastava, A., Madad, S., Lareau, C. A. & Satija, R. Single-cell chromatin state analysis with Signac. *Nat. Methods* **18**, 1333–1341 (2021).

14. Granja, J. M. *et al.* ArchR is a scalable software package for integrative single-cell chromatin accessibility analysis. *Nat. Genet.* **53**, 403–411 (2021).

15. Zhang, Y. *et al.* Model-based analysis of ChIP-Seq (MACS). *Genome Biol.* **9**, R137 (2008).

16. Pedregosa, F. *et al.* Scikit-learn: Machine Learning in Python. *J. Mach. Learn. Res.* **12**, 2825–2830 (2011).

17. Palla, G. *et al.* Squidpy: a scalable framework for spatial omics analysis. *Nat. Methods* **19**, 171–178 (2022).

18. Getis, A. & Ord, J. K. The analysis of spatial association by use of distance statistics. Geogr. Anal. 24, 189–206 (2010).

Supplementary Tables

Supplementary Table S1 Experimental datasets used in the manuscript

Dataset	Name	Platform	Size (spots x genes/ proteins/peaks)	Figure
Dataset1	Mouse spleen replicate1	SPOTS (RNA-protein)	2,568x32,285<br>2,568x21	Figure 4e-l, Extended Data Fig.1g, Extended Data Fig.9, Extended Data Fig.10a-c, Suppl. Figure 10a
Dataset2	Mouse spleen replicate2	SPOTS (RNA-protein)	2,768x32,285<br>2,768x21	Extended Data Fig.10d-h, Suppl. Figure 10b
Dataset3	Mouse Thymus1	Stereo-CITE-seq (RNA-protein)	4,697x23,622<br>4,697x51	Figure 4a-d, Extended Data Fig.8, Suppl. Figure 12a, Suppl. Figure 13
Dataset4	Mouse Thymus2	Stereo-CITE-seq (RNA-protein)	4,253x23,529<br>4,253x19	Suppl. Figure 6, Suppl. Figure 12b, Suppl. Figure 14
Dataset5	Mouse Thymus3	Stereo-CITE-seq (RNA-protein)	4,646x23,960<br>4,646x19	Suppl. Figure 7, Suppl. Figure 12c, Suppl. Figure 15a
Dataset6	Mouse Thymus4	Stereo-CITE-seq (RNA-protein)	4,228x23,221<br>4,228x19	Suppl. Figure 8, Suppl. Figure 12d, Suppl. Figure 15b
Dataset7	Mouse Brain RNA ATAC P22	Spatial-transcriptome-epigenome	9,215x22,914<br>9,215x121,068	Figure 3a-e, Extended Data Fig.1g,h, Extended Data Fig.4a,c,e, Extended Data Fig.5f-h, Suppl. Figure 9d-f
Dataset8	Mouse Brain RNA H3K4me3	Spatial-transcriptome-epigenome	9,548x22,731<br>9,548x35,270	Extended Data Fig.5a-e
Dataset9	Mouse Brain RNA H3K27ac	Spatial-transcriptome-epigenome	9,370x23,415<br>9,370x104,162	Figure 3f-l, Extended Data Fig.4b,d,f, Extended Data Fig.7
Dataset10	Mouse Brain RNA H3K27me3	Spatial-transcriptome-epigenome	9,752x25,881<br>9,752x70,470	Extended Data Fig.6
Dataset11	Human Lymph Node A1	10x Genomics Visium (RNA-protein)	3,484x18,085<br>3,484x31	Figure 2e-i, Extended Data Fig.3a-c
Dataset12	Human Lymph Node D1	10x Genomics Visium (RNA-protein)	3,359x18,085<br>3,359x31	Extended Data Fig.3d-k, Suppl. Figure 11
Dataset13	Simulation 1	NSF (Townes et al., 2023)	1,296x1,000<br>1,296x100	Figure 2a-d, Extended Data Fig.1a-f, Suppl. Figure 1, Suppl. Figure 2a,b, Suppl. Figure 9a-c
Dataset14	Simulation 2	NSF (Townes et al., 2023)	1,296x1,000<br>1,296x100	Suppl. Figure 2c-g
Dataset15	Simulation 3	NSF (Townes et al., 2023)	1,296x1,000<br>1,296x100	Suppl. Figure 3
Dataset16	Simulation 4	NSF (Townes et al., 2023)	1,296x1,000<br>1,296x100	Suppl. Figure 4a-e
Dataset17	Simulation 5	NSF (Townes et al., 2023)	1,296x1,000<br>1,296x100	Suppl. Figure 4f-j
Dataset18	Simulation 6 (triplet omics)	NSF (Townes et al., 2023)	1,296x1,000<br>1,296x100	Extended Data Fig.2

Supplementary Table S2 Applicability of methods

Data type	Seurat	totalVI	MultiVI	MOFA+	MEFISTO	scMM	StabMap	SpatialGlue
RNA & protein	√	√	√	√	√	√	√	√
RNA & ATAC	√	×	√	√	√	√	√	√

Supplementary Table S3 Application of methods on datasets

Datasets	Platform	Seurat	totalVI	MultiVI	MOFA+	MEFISTO	scMM	StabMap	SpatialGlue
Dataset1	SPOTS (RNA-protein)	√	√	√	√	√	√	√	√
Dataset2	SPOTS (RNA-protein)	√	√	√	√	√	√	√	√
Dataset3	Stereo-CITE-seq (RNA-protein)	√	√	√	√	√	√	√	√
Dataset4	Stereo-CITE-seq (RNA-protein)	√	√	√	√	√	√	√	√
Dataset5	Stereo-CITE-seq (RNA-protein)	√	√	√	√	√	√	√	√
Dataset6	Stereo-CITE-seq (RNA-protein)	√	√	√	√	√	√	√	√
Dataset7	Spatial-transcriptome-epigenome	√	×	√	√	×	√	√	√
Dataset8	Spatial-transcriptome-epigenome	√	×	√	√	×	√	√	√
Dataset9	Spatial-transcriptome-epigenome	√	×	√	√	×	√	√	√
Dataset10	Spatial-transcriptome-epigenome	√	×	√	√	×	×	√	√
Dataset11	10x Genomics Visium (RNA-protein)	√	√	√	√	√	√	√	√
Dataset12	10x Genomics Visium (RNA-protein)	√	√	√	√	√	√	√	√
Dataset13	NSF (Townes et al., 2023) (Simulation data, RNA-protein)	√	√	√	√	√	√	√	√
Dataset14	NSF (Townes et al., 2023) (Simulation data, RNA-protein)	√	√	√	√	√	√	√	√
Dataset15	NSF (Townes et al., 2023) (Simulation data, RNA-protein)	√	√	√	√	√	√	√	√
Dataset16	NSF (Townes et al., 2023) (Simulation data, RNA-protein)	√	√	√	√	√	√	√	√
Dataset17	NSF (Townes et al., 2023) (Simulation data, RNA-protein)	√	√	√	√	√	√	√	√

Supplementary Table S4. Summary of simulation parameters. Here, “pi” denotes the zero-inflation probability of the ZINB (Zero-Inflated Negative Binomial) distribution, “nzprob_nsp” denotes the probability of a “one” (else zero) for nonspatial factors, and “bkg_mean” denotes the negative binomial mean for observations that are “zero” in the factors. “mean” and “std” are mean and standard deviation of the Gaussian distribution, respectively.

Dataset	Modality 1						Modality 2				
	ZINB			Gaussian		dimension	NB		Gaussian		dimension
	pi	nzprob_nsp	bkg_mean	mean	std		nzprob_nsp	bkg_mean	mean	std	
Simulation 1	0.5	0.2	0.2	2	0.5	1,000	0.25	0.4	2	0.5	100
Simulation 2	0.5	0.2	0.3	2	0.5	1,000	0.25	0.5	2	0.5	100
Simulation 3	0.5	0.2	0.4	2	0.5	1,000	0.25	0.6	2	0.5	100
Simulation 4	0.5	0.2	0.5	2	0.5	1,000	0.25	0.7	2	0.5	100
Simulation 5	0.5	0.2	0.6	2	0.5	1,000	0.25	0.8	2	0.5	100

Supplementary Table S5. Summary of technical specifications of different technologies.

Platform	Spatial resolution (μm)	Distance between spots (μm)	Image area size
10x Genomics Visium & SPOTS	55	100	6.5 x 6.5 mm
Stereo-seq	0.22	0.5	200 mm^2
Spatial-epigenome-transcriptome	20	-	50x50 or 100x100 grid

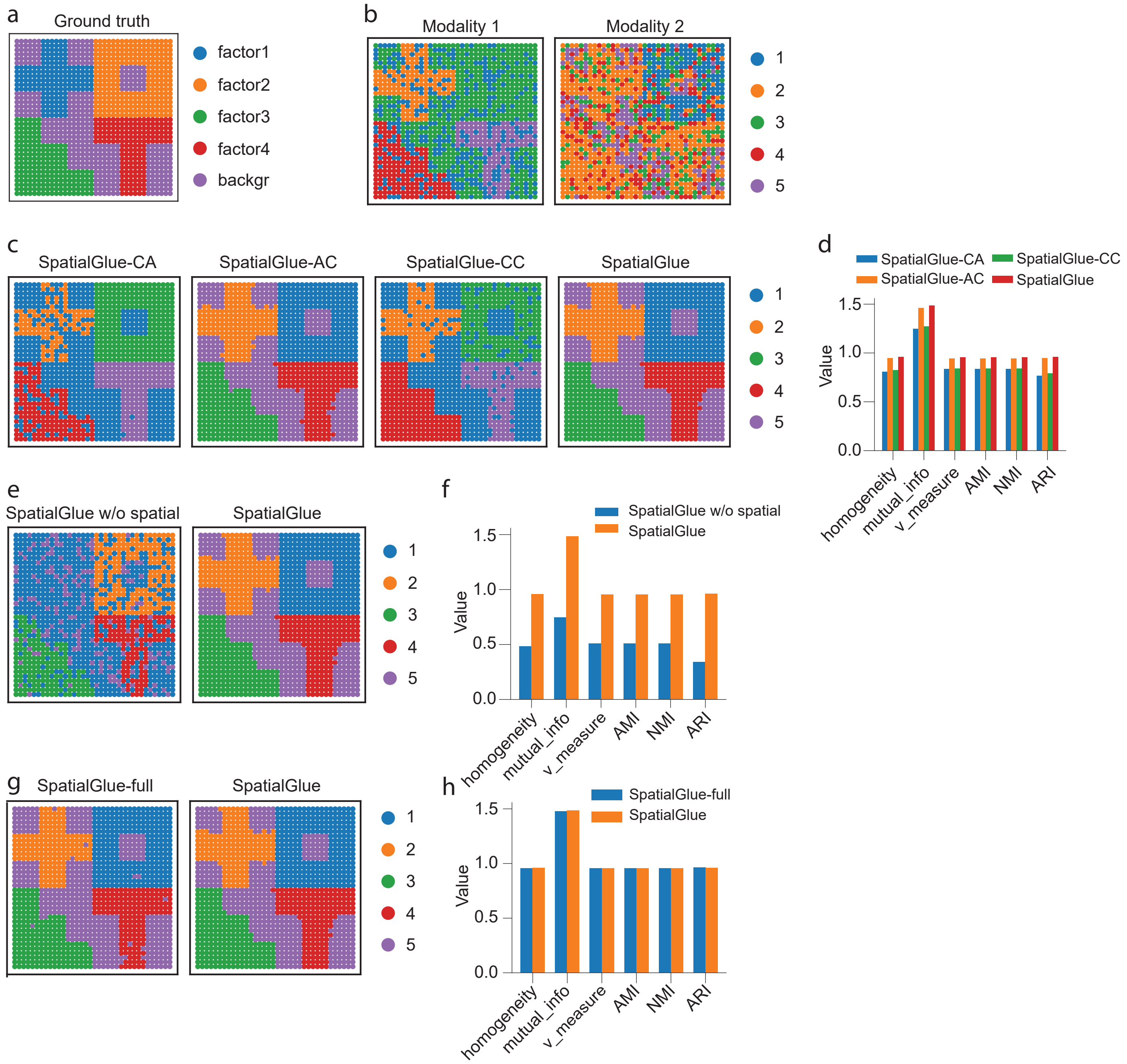

Supplementary Figure S1. Ablation study to validate the contribution of each component to the performance of the SpatialGlue model. The ablation study was conducted using the simulated data 1. (a) Ground truth. (b) Spatial clustering of modalities 1 and 2. (c) Comparison of SpatialGlue and its variants, i.e., using concatenation (C) instead of attention (A) for intra-modality integration (SpatialGlue-CA), using concatenation instead of attention inter-modality for integration (SpatialGlue-AC), and using concatenation instead of attention for both intra- and inter-modality integration (SpatialGlue-CC). (d) Quantitative evaluation of SpatialGlue and variants (CA, AC, CC) with the six supervised metrics. (e) Clustering results of SpatialGlue and the non-spatial variant (SpatialGlue w/o spatial). (f) Quantitative comparison of SpatialGlue with 'SpatialGlue w/o spatial'. (g) Comparison of SpatialGlue

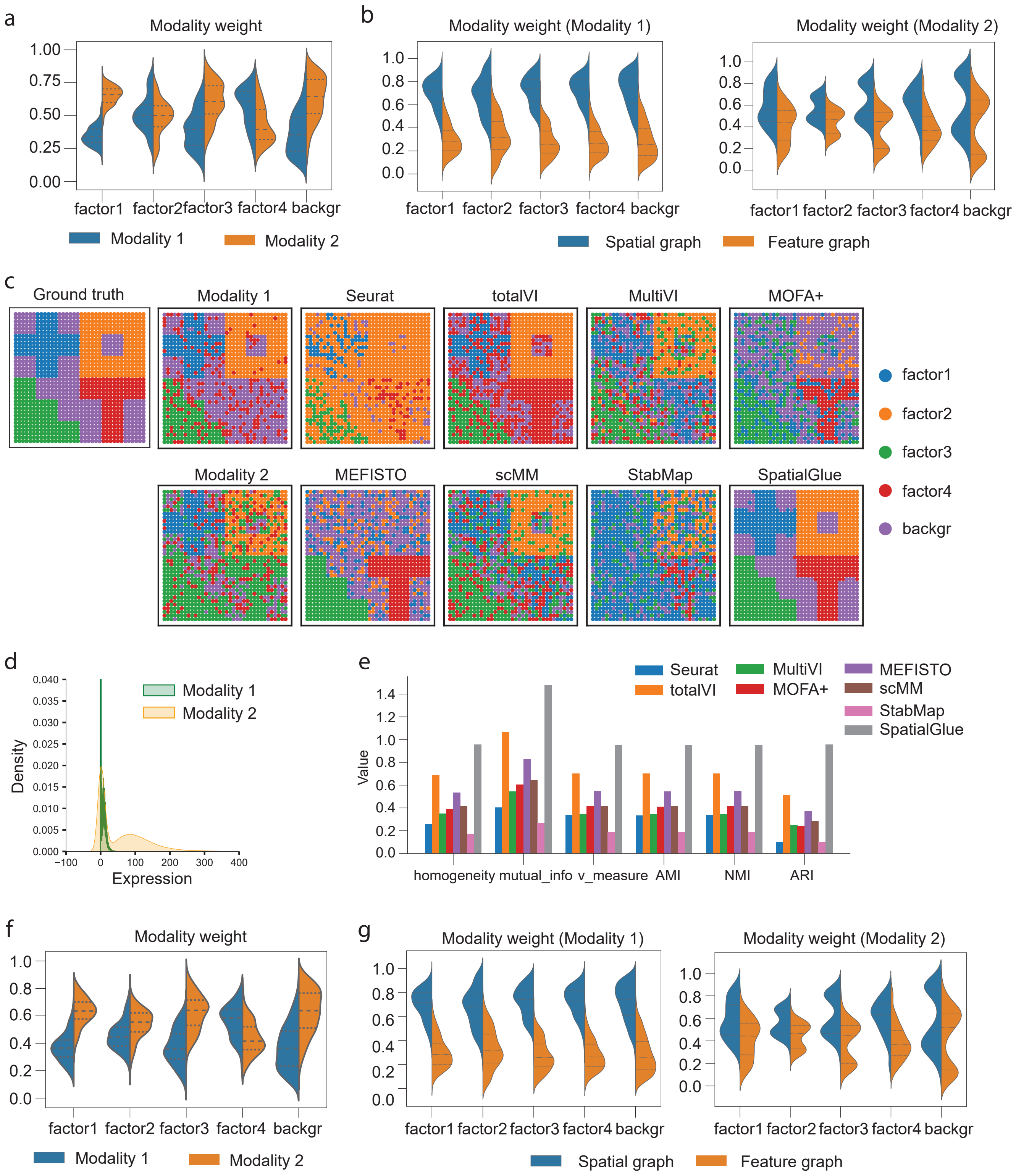

Supplementary Figure S2: Results for simulation data 1 and 2. (a) Modality weights of different modalities, denoting their importance to the integrated output of SpatialGlue with simulation data 1. (b) SpatialGlue's within-modality weights for the importance of spatial and feature graphs with simulation data 1. (c) Simulated data 2 ground truth, unimodal clustering of modalities, and integration results from Seurat, totalVI, MultiVI, MOFA+, MEFISTO, scMM, StabMap, and SpatialGlue. (d) Density distribution of the simulated data modalities. (e) Quantitative comparison of the eight methods with six measurement metrics, homogeneity, mutual information, V measure score, adjusted mutual information (AMI), normalized mutual information (NMI), and adjusted rand index (ARI). (f) SpatialGlue's between-modality weights explaining the importance of each modality to each cluster. (g) Within-modality weights for the importance of spatial and feature graphs.

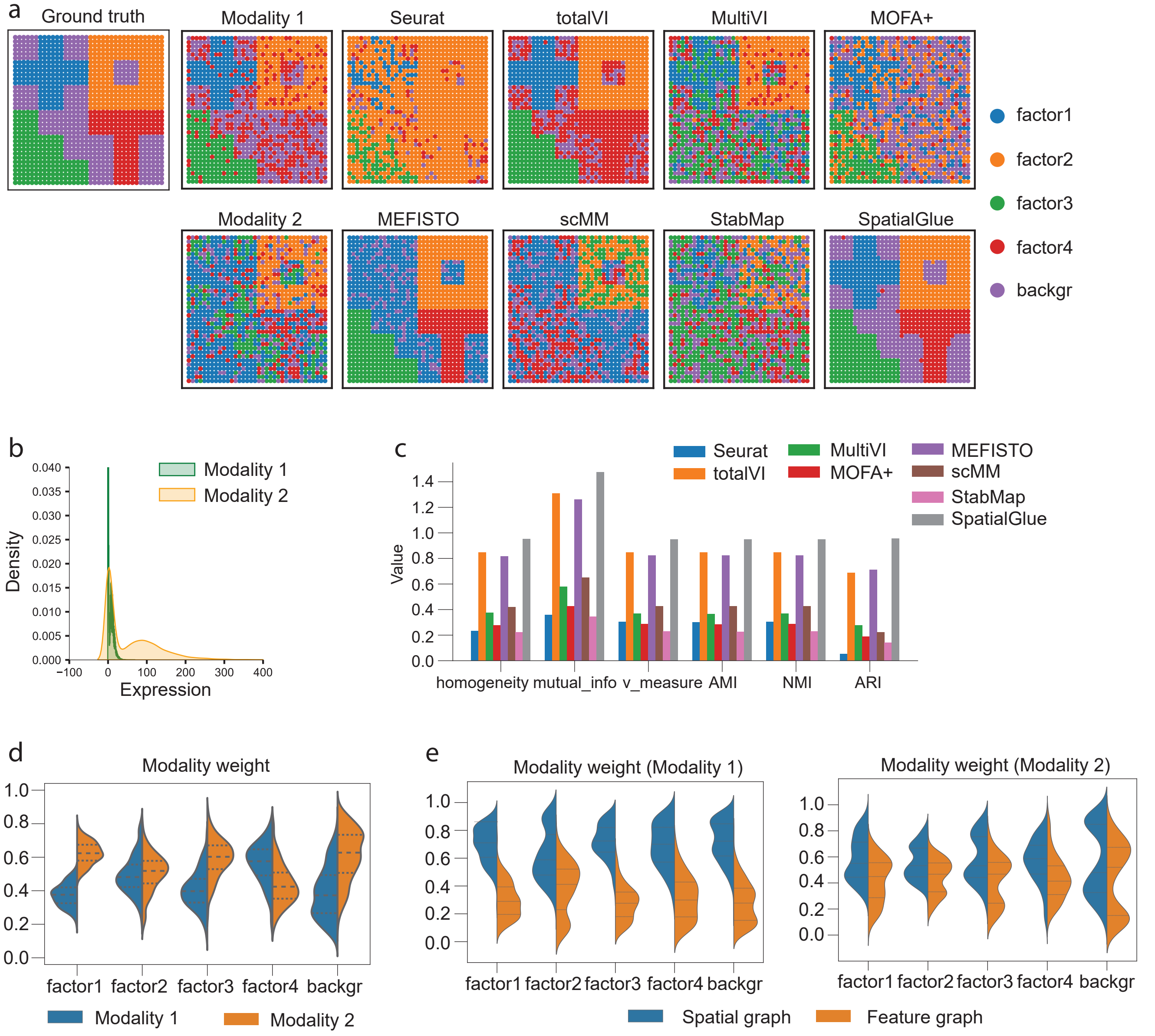

Supplementary Figure S3. Results for simulation data 3. (a) Modality weights of different modalities, denoting their importance to the integrated output of SpatialGlue with simulation data 1. (b) SpatialGlue's within-modality weights for the importance of spatial and feature graphs with simulation data 1. (c) Simulated data 2 ground truth, unimodal clustering of modalities, and integration results from Seurat, totalVI, MultiVI, MOFA+, MEFISTO, scMM, StabMap, and SpatialGlue. (d) Density distribution of the simulated data modalities. (e) Quantitative comparison of the eight methods with six measurement metrics, homogeneity, mutual information, V measure score, adjusted mutual information (AMI), normalized mutual information (NMI), and adjusted rand index (ARI). (f) SpatialGlue's between-modality weights explaining the importance of each modality to each cluster. (g) Within-modality weights for the importance of spatial and feature graphs.

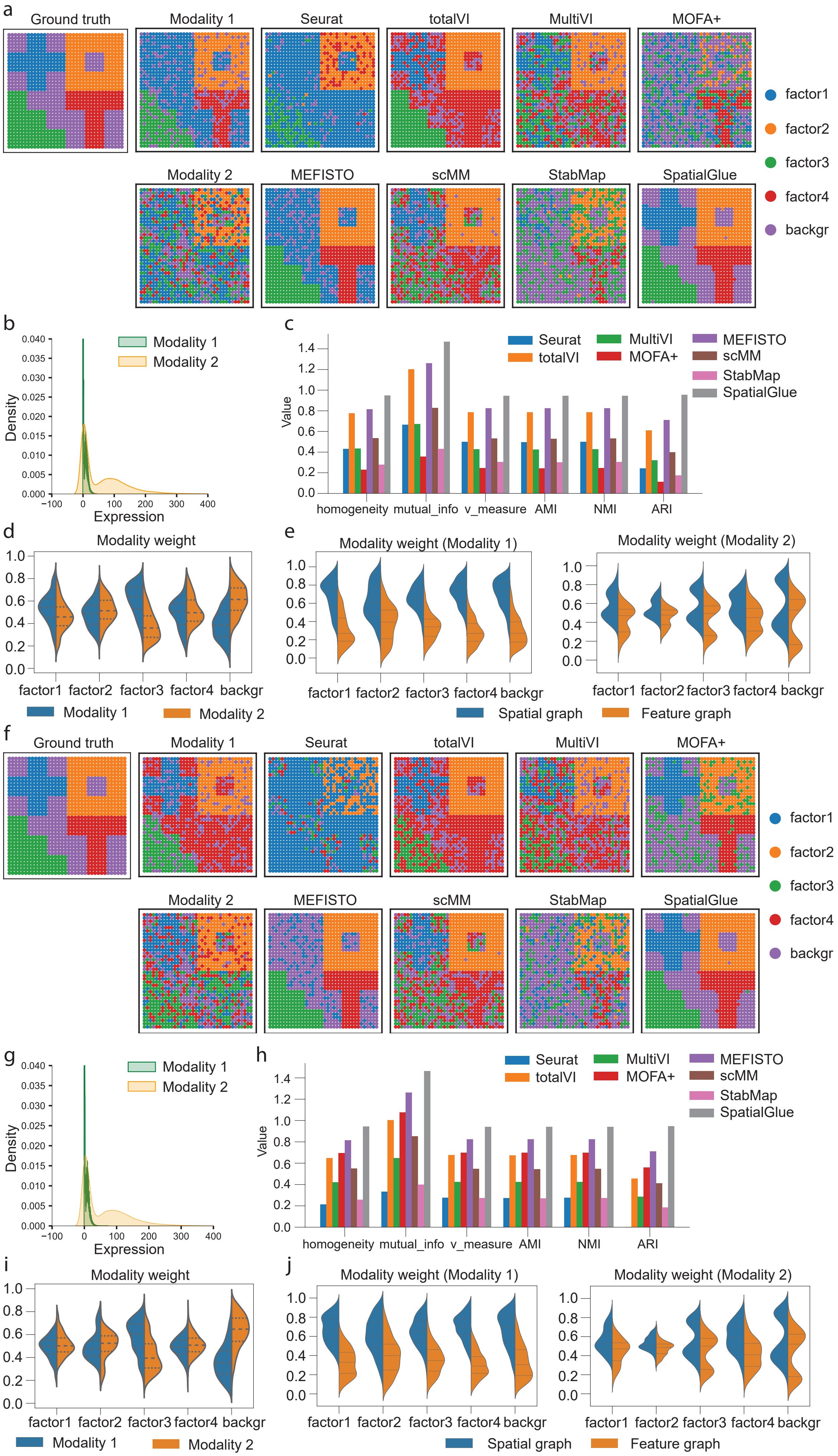
a
Ground truth
Modality 1
Seurat
totalVI
MultiVI
MOFA+
Modality 2
MEFISTO
scMM
StabMap
SpatialGlue
factor1
factor2
factor3
factor4
backgr
b
Modality 1
Modality 2
Density
Expression
c
Seurat
totalVI
MultiVI
MOFA+
MEFISTO
scMM
StabMap
SpatialGlue
Value
homogeneity
mutual_info
v_measure
AMI
NMI
ARI
d
Modality weight
factor1
factor2
factor3
factor4
backgr
Modality 1
Modality 2
e
Modality weight (Modality 1)
Modality weight (Modality 2)
Spatial graph
Feature graph
f
g
h
i
j

Supplementary Figure S4. Results for simulation data 4 and 5. (a) Simulated data 4 ground truth, unimodal clustering of modalities, and integration results from Seurat, totalVI, MultiVI, MOFA+, MEFISTO, scMM, StabMap, and SpatialGlue. (b) Density distribution of the simulated data modalities. (c) Quantitative comparison of the eight methods with six measurement metrics, homogeneity, mutual information, V measure score, adjusted mutual information (AMI), normalized mutual information (NMI), and adjusted rand index (ARI). (d) SpatialGlue's between-modality weights explaining the importance of each modality to each cluster. (e) Within-modality weights for the importance of spatial and feature graphs. (f) Simulated data 5 ground truth, unimodal clustering of modalities, and integration results from Seurat, totalVI, MultiVI, MOFA+, MEFISTO, scMM, StabMap, and SpatialGlue. (g) Density distribution of the simulated data modalities. (h) Quantitative comparison of the eight methods with six measurement metrics, homogeneity, mutual information, V measure score, adjusted mutual information (AMI), normalized mutual information (NMI), and adjusted rand index (ARI). (i) SpatialGlue's between-modality weight explaining the importance of each modality to each cluster. (j) Within-modality weights for the importance of spatial and feature graphs.

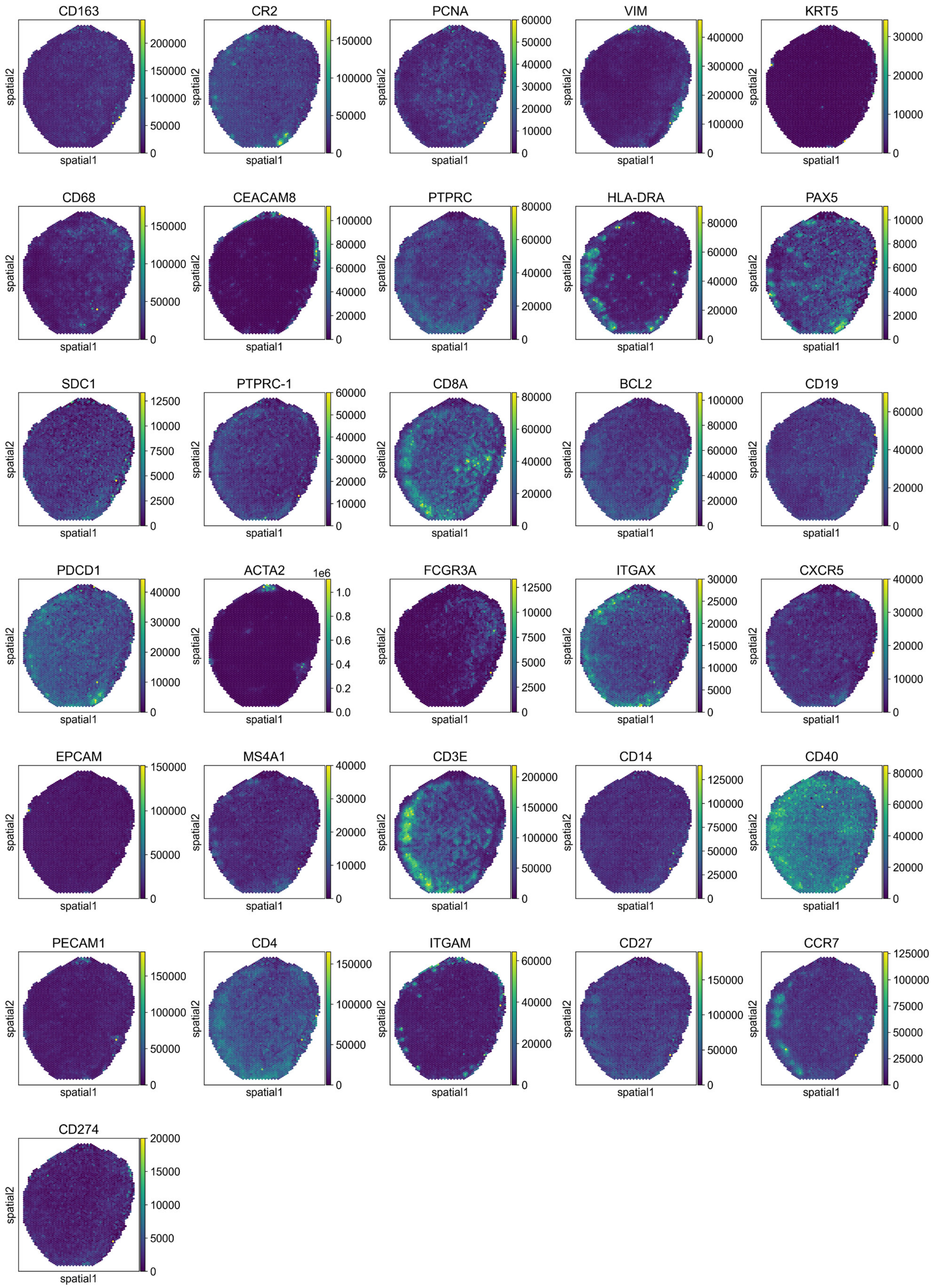

Supplementary Figure S5. ADT intensity plots of the lymph node A1 sample.

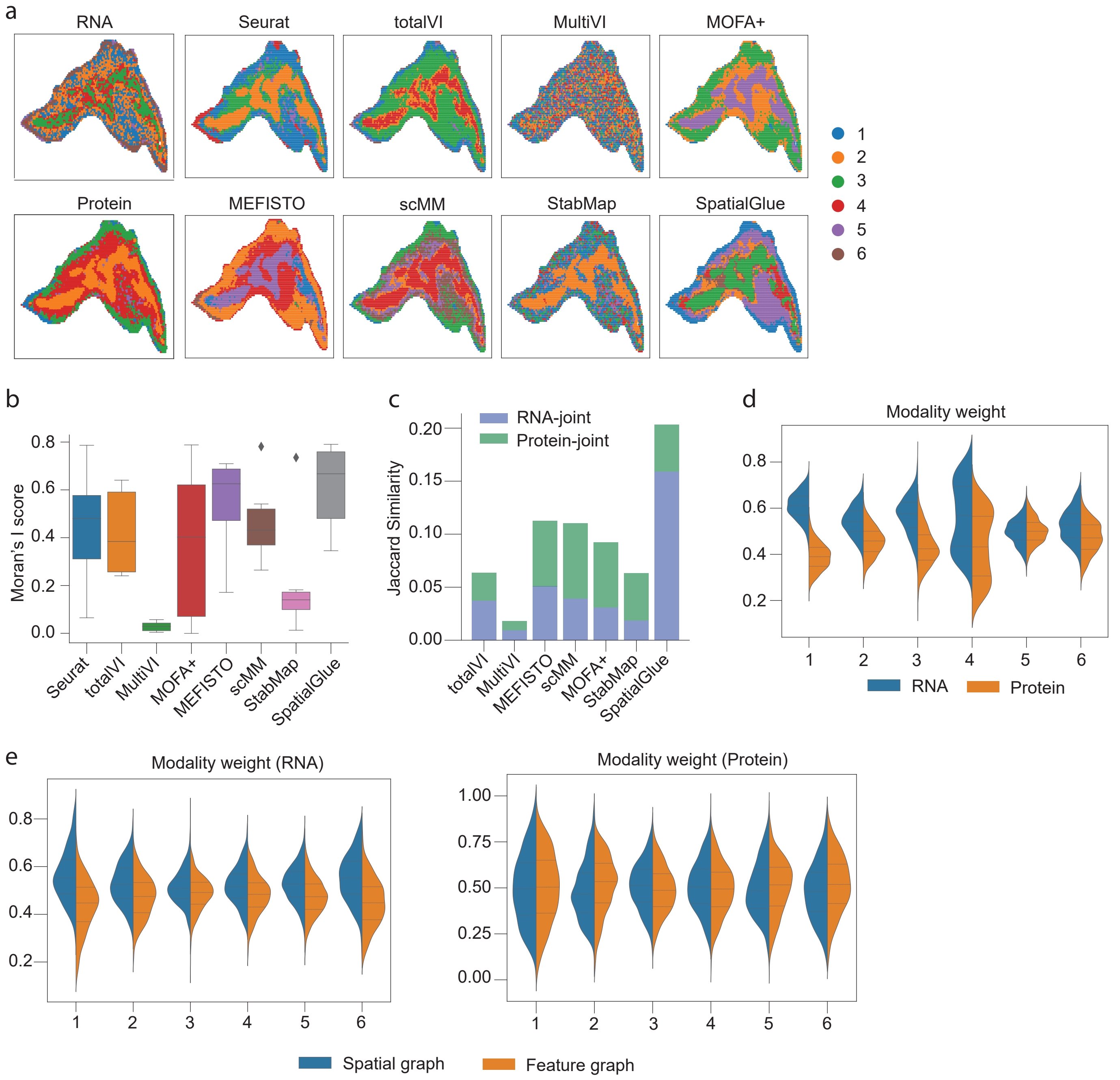

Supplementary Figure S6. Results for the mouse thymus 2 sample. (a) Spatial plots of data modalities with unimodal clustering (left), and clustering results (right) from single-cell and spatial multi-omics integration methods, Seurat, totalVI, MultiVI, MOFA+, MEFISTO, scMM, StabMap, and SpatialGlue. (b) Comparison of Moran's I score. In the boxplot, the center line denotes the median, box limits denote the upper and lower quartiles, and whiskers denote the 1.5× interquartile range. n=6 clusters. (c) Comparison of Jaccard Similarity scores. (d) Between-modality weight explaining the importance of each modality to each cluster. (e) Within-modality weights explaining the contributions of the spatial and feature graphs to each cluster for each modality.

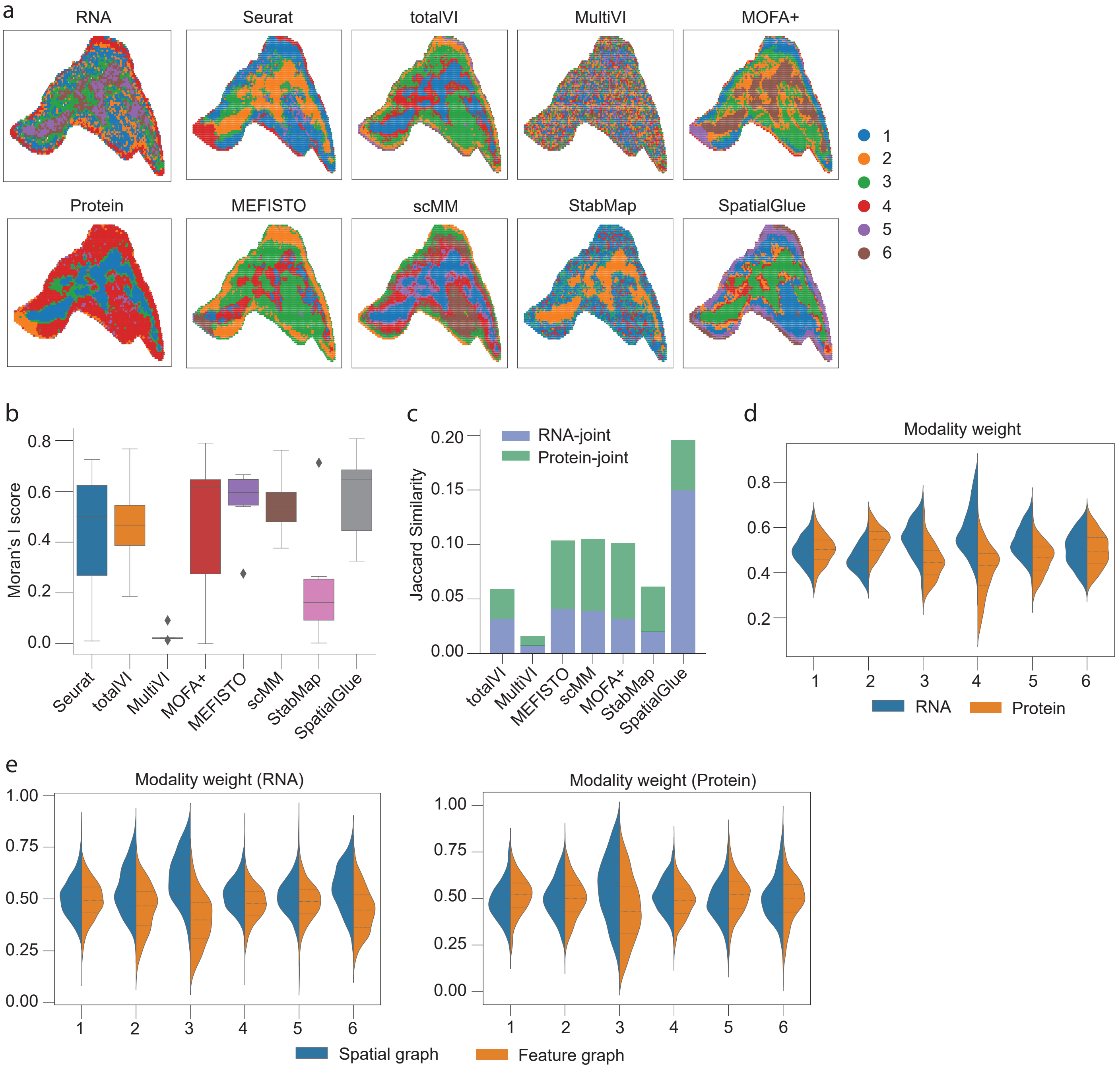

Supplementary Figure S7. Results for the mouse thymus 3 sample. (a) Spatial plots of data modalities with unimodal clustering (left), and clustering results (right) from single-cell and spatial multi-omics integration methods, Seurat, totalVI, MultiVI, MOFA+, MEFISTO, scMM, StabMap, and SpatialGlue. (b) Comparison of Moran's I score. In the boxplot, the center line denotes the median, box limits denote the upper and lower quartiles, and whiskers denote the 1.5× interquartile range. n=6 clusters. (c) Comparison of Jaccard Similarity scores. (d) Between-modality weight explaining the importance of each modality to each cluster. (e) Within-modality weights

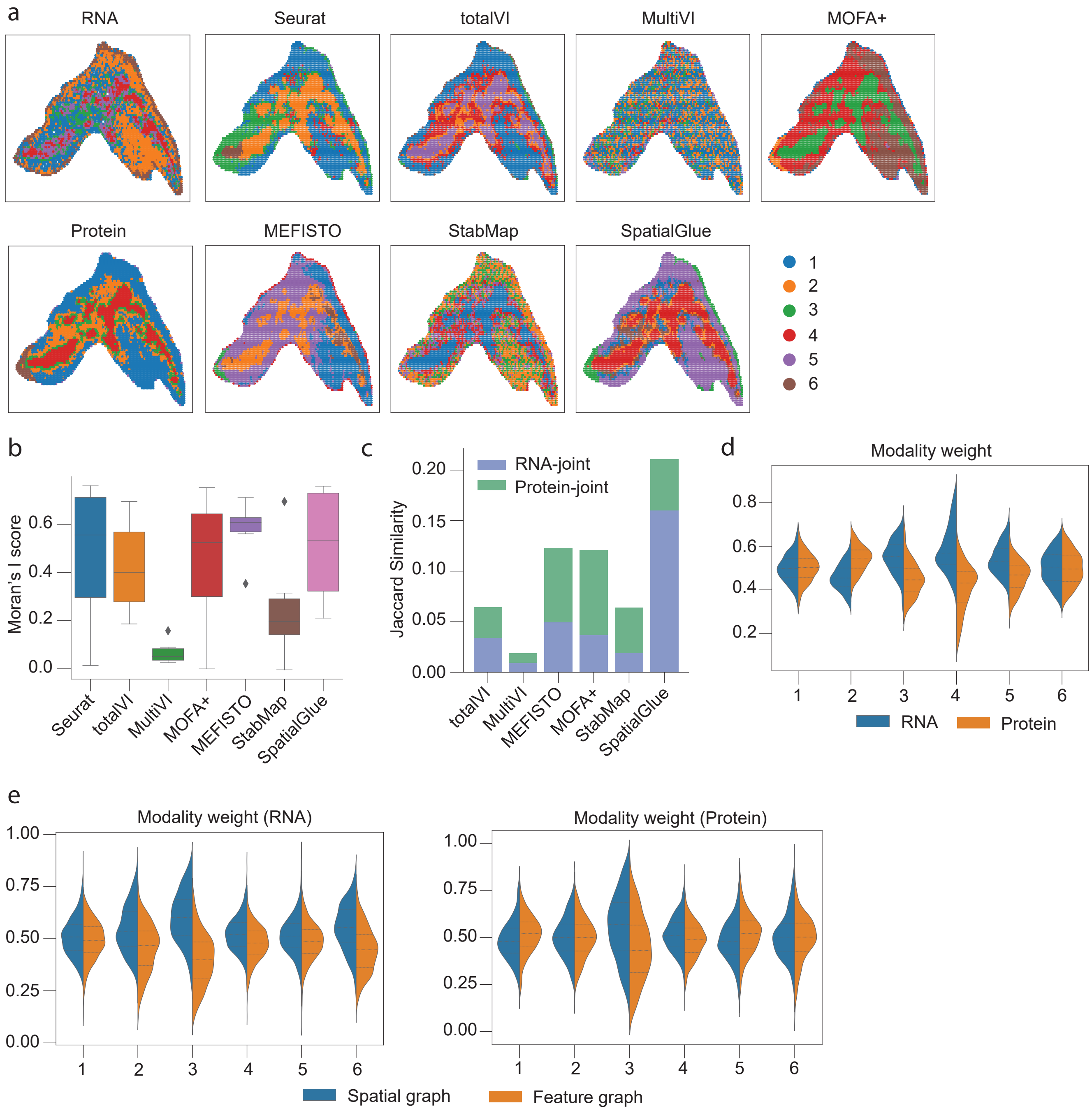

Supplementary Figure S8. Results for the mouse thymus 4 sample. (a) Spatial plots of data modalities with unimodal clustering (left), and clustering results (right) from single-cell and spatial multi-omics integration methods, Seurat, totalVI, MultiVI, MOFA+, MEFISTO, StabMap, and SpatialGlue. (b) Comparison of Moran's I score. In the boxplot, the center line denotes the median, box limits denote the upper and lower quartiles, and whiskers denote the 1.5× interquartile range. n=6 clusters. (c) Comparison of Jaccard Similarity scores. (d) Between-modality weight explaining the importance of each modality to each cluster. (e) Within-modality weights explaining

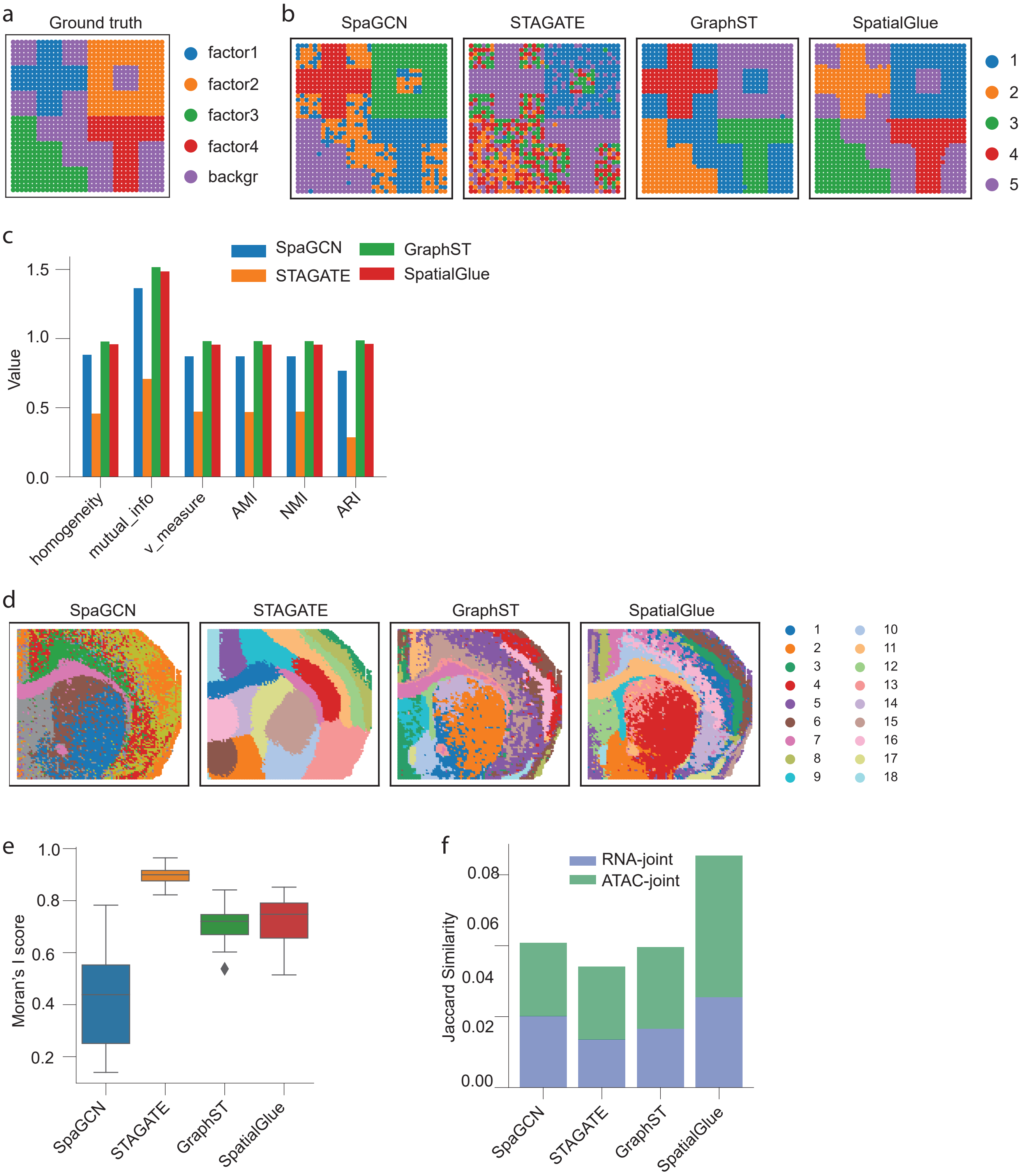

Supplementary Figure S9. Comparison between SpatialGlue and single-modal methods on simulated and real (mouse brain P22 sample acquired using spatial-ATAC-RNA-seq) data. (a) Ground truth of the simulated data. (b) Comparison between SpatialGlue and single-modal methods, SpaGCN, STAGATE, and GraphST on the simulated data. (c) Quantitative evaluation using six supervised metrics. (d) Comparison between SpatialGlue and single-modal methods on the mouse brain P22 sample data. (e) Comparison of Moran's I score. In the boxplot, the center line denotes the median, box limits denote the upper and lower quartiles, and whiskers denote the 1.5× interquartile range. n=18 clusters. (f) Comparison of Jaccard Similarity scores.

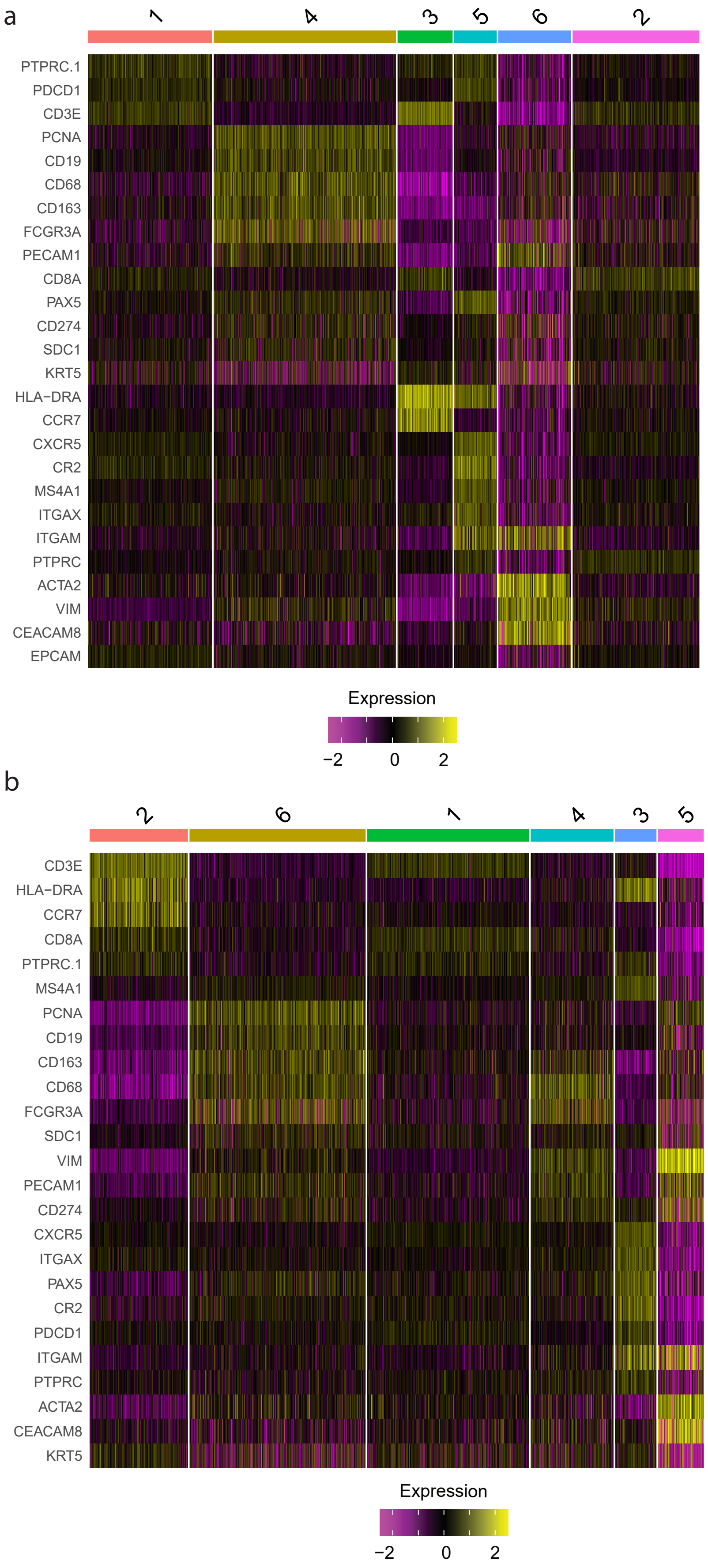

Supplementary Figure S10: Heatmap of differentially expressed ADTs for each cluster for the human lymph node A1 (a) and D1 (b) samples.

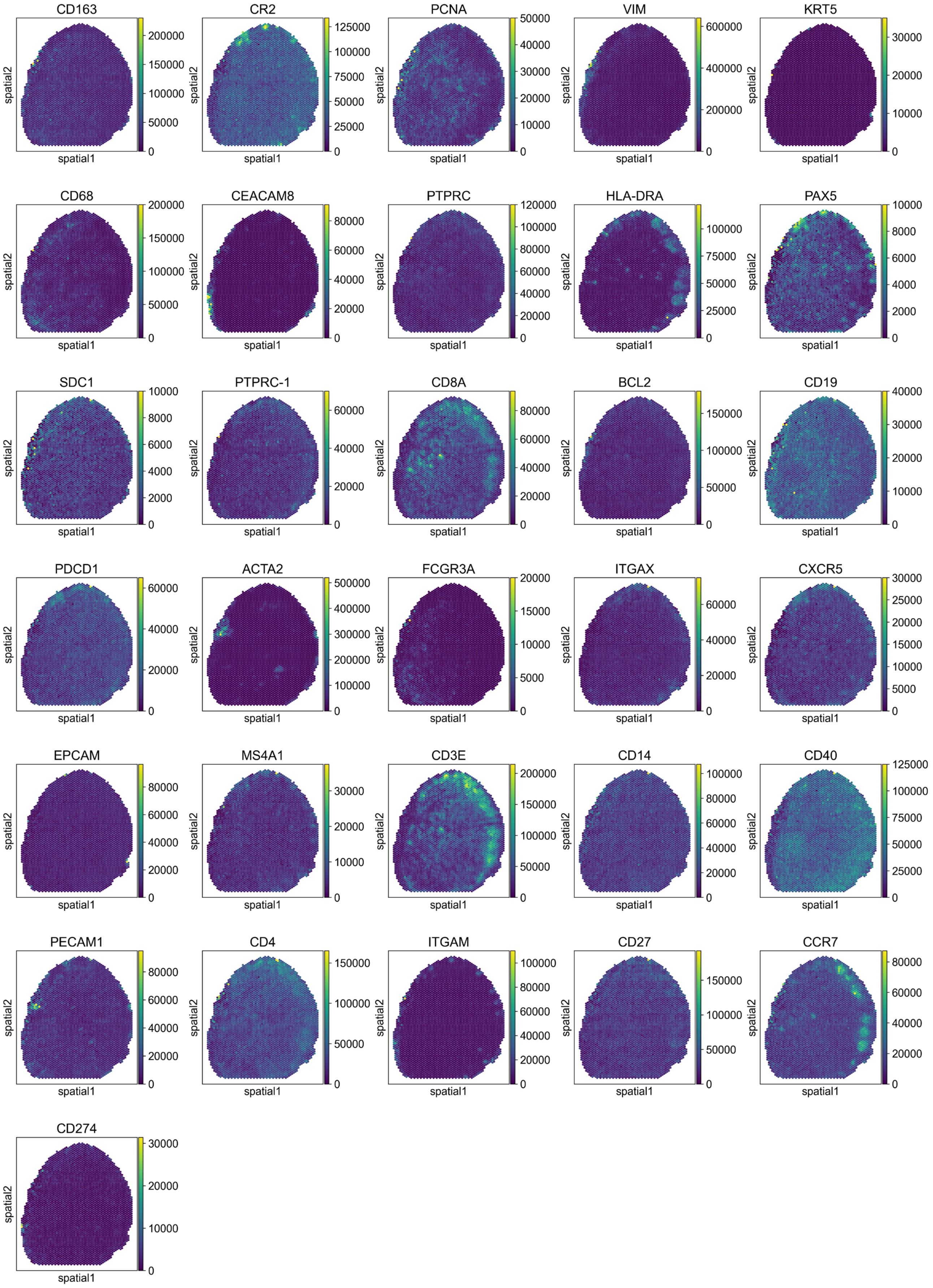

Supplementary Figure S11. ADTs intensity plots of the lymph node D1 sample.

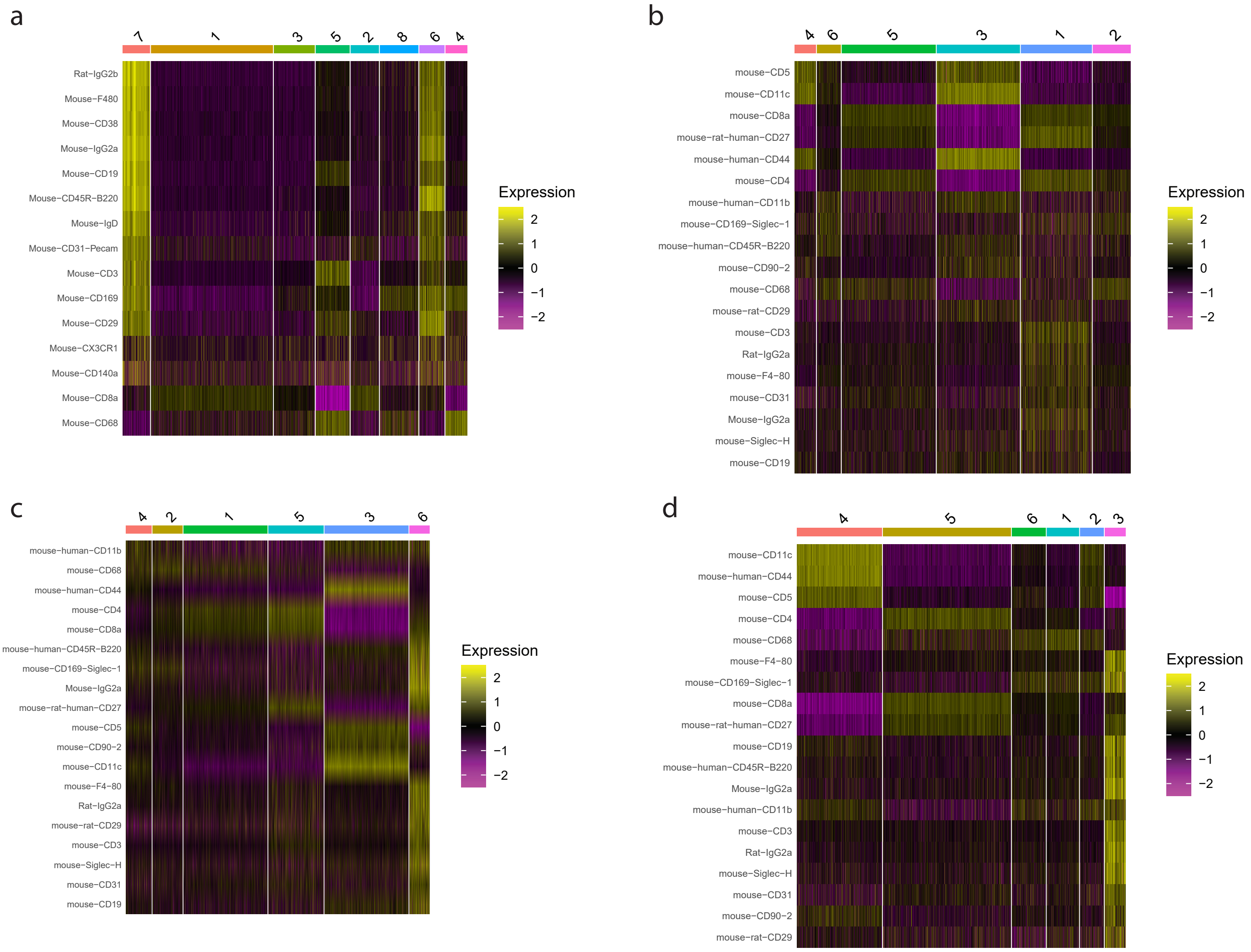

Supplementary Figure S12. Heatmap of differentially expressed ADTs for each cluster from the mouse thymus 1 (a), 2 (b), 3 (c), and 4 (d) samples.

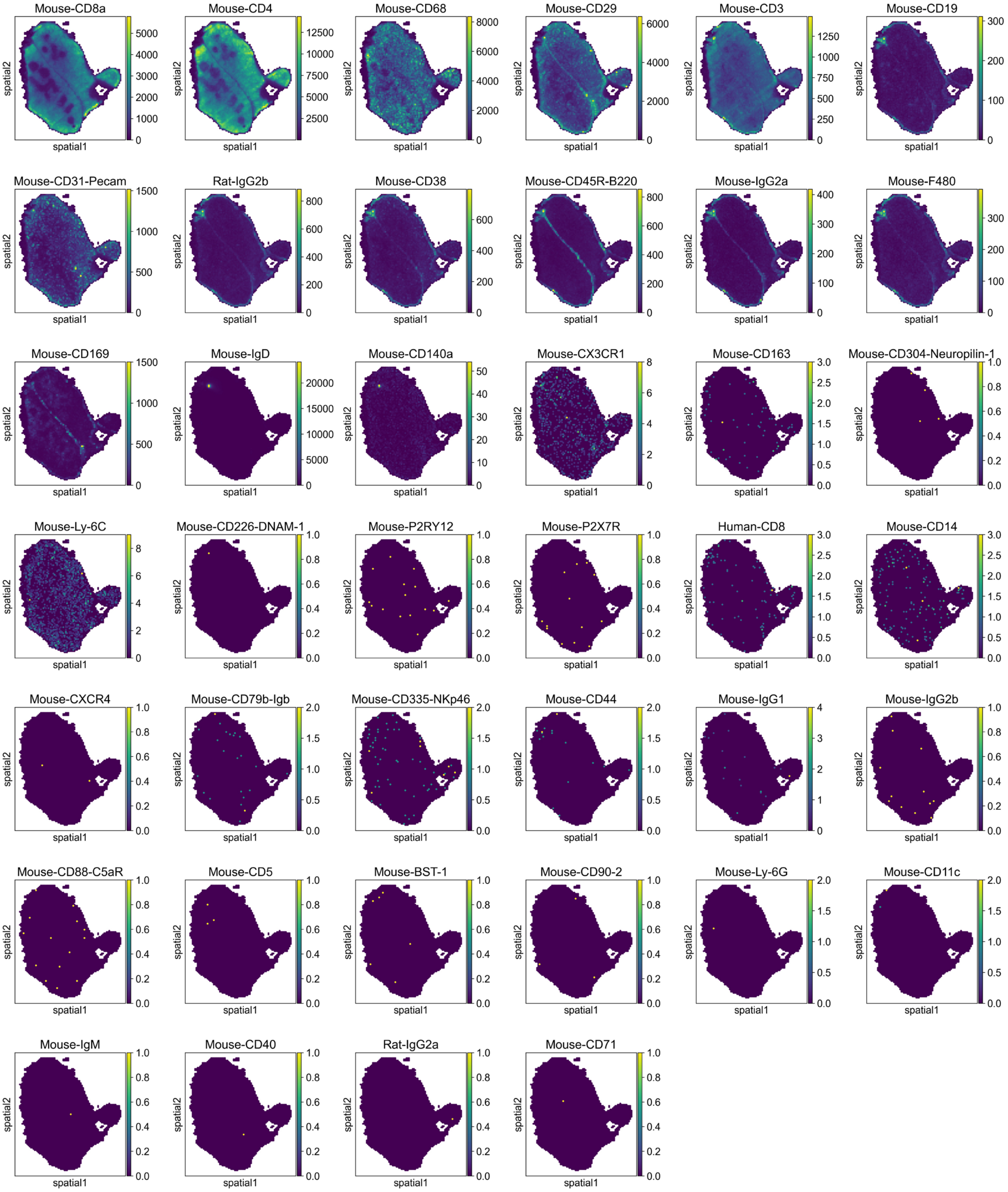

Supplementary Figure S13. Intensity plots of ADTs for the mouse thymus 1 sample.

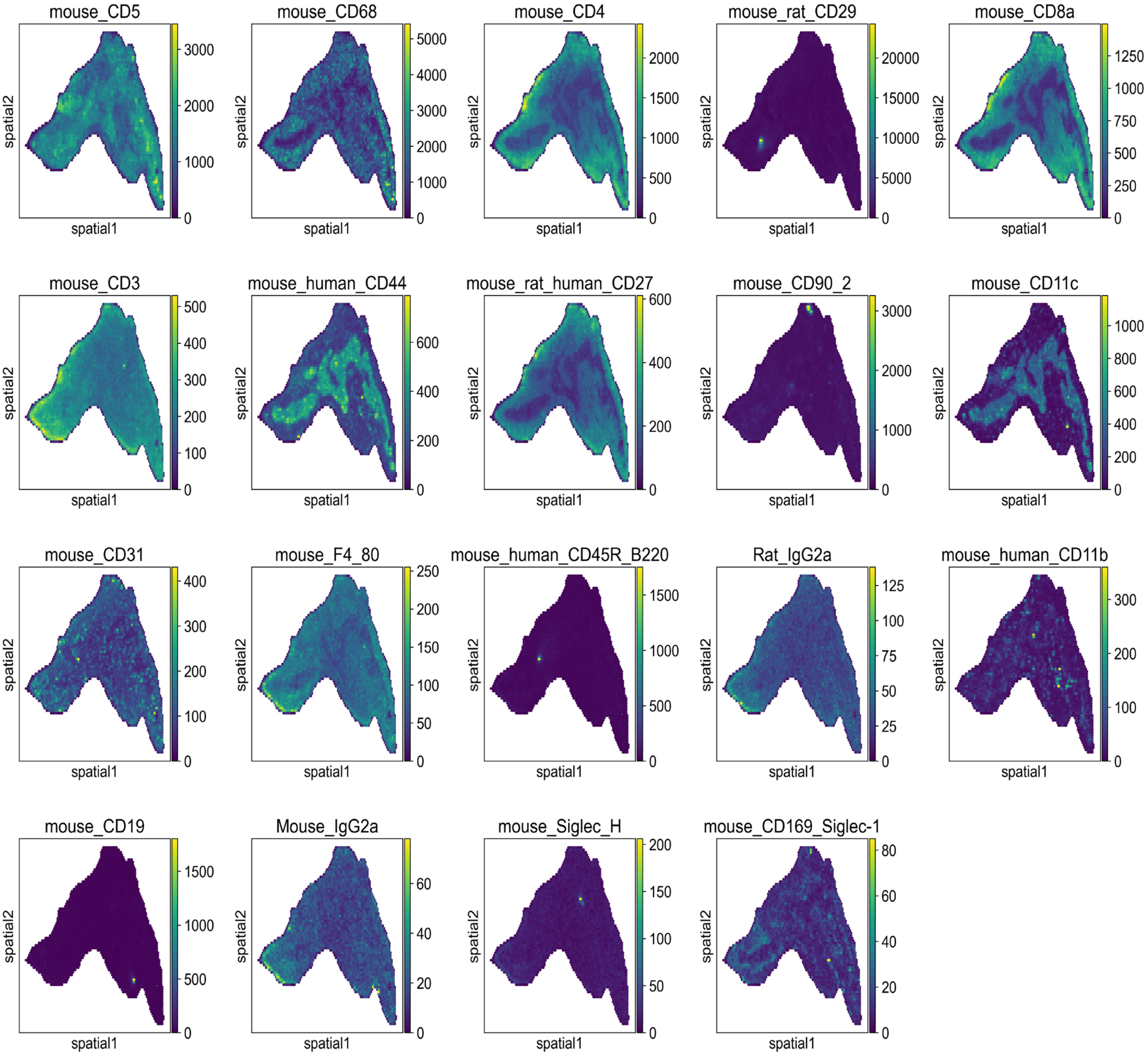

Supplementary Figure S14. Intensity plots of ADTs for the mouse thymus 2 sample.

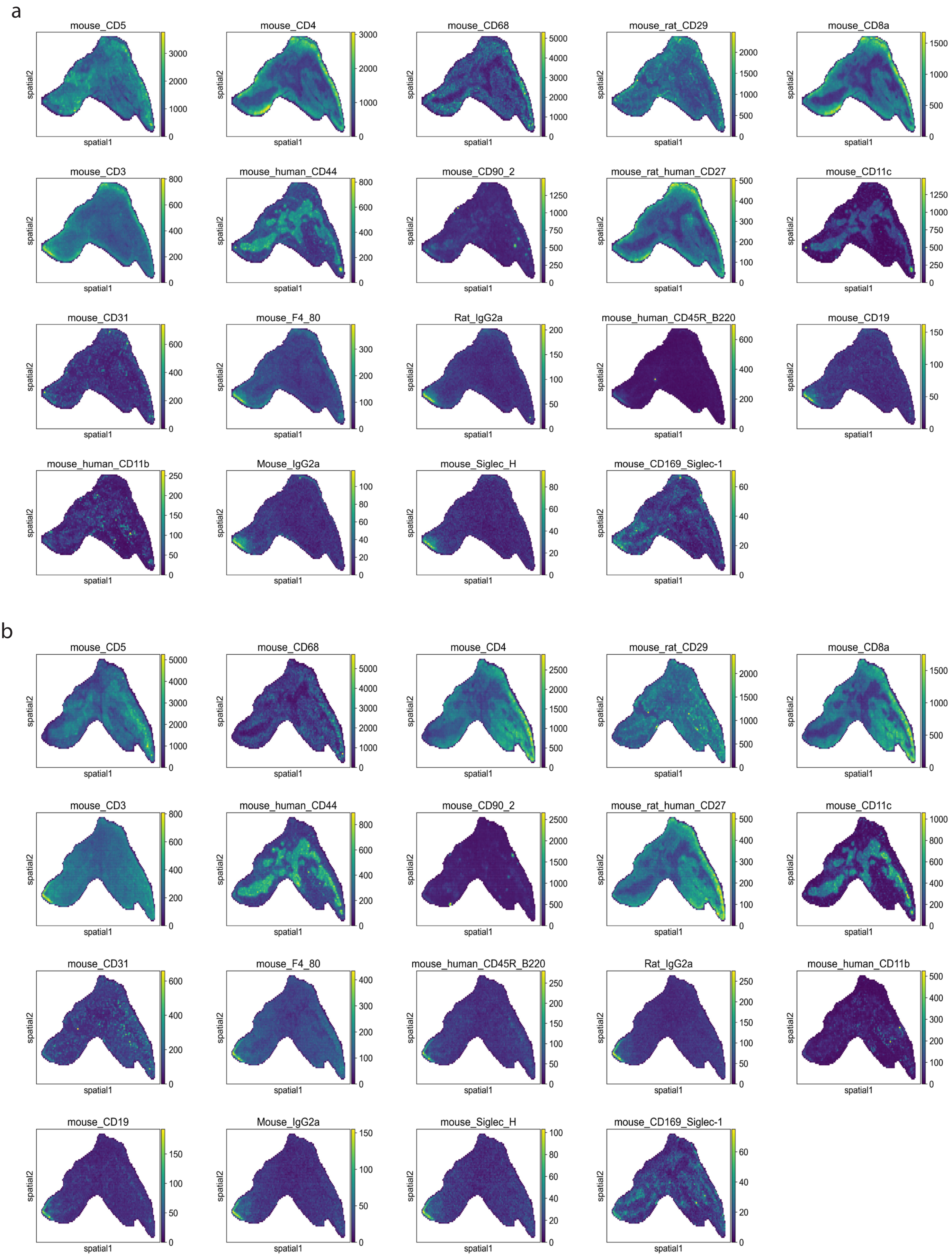

Supplementary Figure S15. Intensity plots of ADTs for the mouse thymus 3 (a) and 4 (b) samples.
