# Supplementary material for: Deciphering spatial domains from spatial multi-omics with SpatialGlue
Source: Nat Methods. 2024 Jun 21;21(9):1658–67. doi: 10.1038/s41592-024-02316-4 (PMC11399094; doi:10.1038/s41592-024-02316-4)
Supplement: Supplementary file 2 — Reporting Summary [file 41592_2024_2316_MOESM2_ESM.pdf]

nature portfolio

Corresponding author(s): Jinmiao Chen

Last updated by author(s): 26 March, 2024

Reporting Summary

Nature Portfolio wishes to improve the reproducibility of the work that we publish. This form provides structure for consistency and transparency in reporting. For further information on Nature Portfolio policies, see our Editorial Policies and the Editorial Policy Checklist.

Statistics

For all statistical analyses, confirm that the following items are present in the figure legend, table legend, main text, or Methods section.

n/a	Confirmed	
☐	☒	The exact sample size (*n*) for each experimental group/condition, given as a discrete number and unit of measurement
☐	☒	A statement on whether measurements were taken from distinct samples or whether the same sample was measured repeatedly
☒	☐	The statistical test(s) used AND whether they are one- or two-sided<br>*Only common tests should be described solely by name; describe more complex techniques in the Methods section.*
☐	☒	A description of all covariates tested
☐	☒	A description of any assumptions or corrections, such as tests of normality and adjustment for multiple comparisons
☐	☒	A full description of the statistical parameters including central tendency (e.g. means) or other basic estimates (e.g. regression coefficient) AND variation (e.g. standard deviation) or associated estimates of uncertainty (e.g. confidence intervals)
☒	☐	For null hypothesis testing, the test statistic (e.g. *F*, *t*, *r*) with confidence intervals, effect sizes, degrees of freedom and *P* value noted<br>*Give P values as exact values whenever suitable.*
☒	☐	For Bayesian analysis, information on the choice of priors and Markov chain Monte Carlo settings
☐	☒	For hierarchical and complex designs, identification of the appropriate level for tests and full reporting of outcomes
☒	☐	Estimates of effect sizes (e.g. Cohen's *d*, Pearson's *r*), indicating how they were calculated

Our web collection on statistics for biologists contains articles on many of the points above.

Software and code

Policy information about availability of computer code

Data collection	No software was used for data collection.
Data analysis	Seurat v4.0.0 (https://github.com/satijalab/seurat), scvi-tools v1.0.2 (https://github.com/scverse/scvi-tools), MOFA+ v1.9.2 (https://github.com/bioFAM/MOFA2), MEFISTO v1.13.0 (https://github.com/bioFAM/MEFISTO_analyses), scMM (version unavailable) (https://github.com/kodaim1115/scMM), StabMap (version unavailable) (https://github.com/MarioniLab/StabMap), and SpatialGlue v1.1.5 (https://github.com/JinmiaoChenLab/SpatialGlue) were used for integrating spatial multi-omics data. Scanpy v1.9.1 was used for data pre-processing and result visualization. mclust v6.0.0 was used for result visualization. Signac v1.12.0 (https://github.com/stuart-lab/signac) and ArchR v1.0.2 (https://github.com/GreenleafLab/ArchR) were used for downstream analysis for spatial-epigenome-transcriptome data. Space Ranger v2.1.0 (https://www.10xgenomics.com/support/software/space-ranger/latest), MACS2 v2.2.6 (https://github.com/hbctraining/Intro-to-ChIPseq), and Loupe Brower v8.0 (https://www.10xgenomics.com/support/software/loupe-browser/latest) were used for generating in-house data.

For manuscripts utilizing custom algorithms or software that are central to the research but not yet described in published literature, software must be made available to editors and reviewers. We strongly encourage code deposition in a community repository (e.g. GitHub). See the Nature Portfolio guidelines for submitting code & software for further information.

Data

Policy information about availability of data

All manuscripts must include a data availability statement. This statement should provide the following information, where applicable:

- Accession codes, unique identifiers, or web links for publicly available datasets
- A description of any restrictions on data availability
- For clinical datasets or third party data, please ensure that the statement adheres to our policy

We analyzed 12 spatial multi-omics datasets across different data types and technology platforms, including 2 mouse spleen datasets acquired with SPOTS (Ben-Chetrit et al., 2023), 4 mouse thymus datasets from Stereo-CITE-seq (unpublished), and 4 mouse brain spatial-epigenome-transcriptome datasets (Zhang et al. 2023), and the 2 in-house human lymph node datasets acquired with 10x Visium CytAssist.

The SPOTS mouse spleen data was obtained from the GEO repository (accession no. GSE198353, https://www.ncbi.nlm.nih.gov/geo/query/acc.cgi?acc=GSE198353), the Stereo-CITE-seq mouse thymus data from BGI and the spatial-epigenome-transcriptome mouse brain data from AtlasXplore (https://web.atlasxomics.com/visualization/Fan). GRCh38.p13 human genome was obtained from the GENCODE repository (accession no. GENCODE v32/Ensembl 98, https://www.gencodegenes.org/human/release_32.html). The details of all datasets used are available in the Methods section. The data used as input to the methods tested in this study, inclusive of the Stereo-CITE-seq and the in-house human lymph node data have been uploaded to Zenodo and is freely available at https://zenodo.org/record/7879713#.ZE3aOnZByUk.

We have added a 'data availability' section in the manuscript.

Human research participants

Policy information about studies involving human research participants and Sex and Gender in Research.

Reporting on sex and gender	N.A.
Population characteristics	N.A.
Recruitment	N.A.
Ethics oversight	N.A.

Note that full information on the approval of the study protocol must also be provided in the manuscript.

Field-specific reporting

Please select the one below that is the best fit for your research. If you are not sure, read the appropriate sections before making your selection.

☒ Life sciences ☐ Behavioural & social sciences ☐ Ecological, evolutionary & environmental sciences

For a reference copy of the document with all sections, see nature.com/documents/nr-reporting-summary-flat.pdf

Life sciences study design

All studies must disclose on these points even when the disclosure is negative.

Sample size	We used 10 publicly available data and 2 in-house data in the manuscript. For spatial transcriptomics analysis of human lymph node tissues, two sequential sections of 5 µm thickness were utilized from formalin-fixed, paraffin-embedded (FFPE) lymph node.
Data exclusions	We did not remove any spots from the spatial transcriptomic datasets. We also did not remove specific genes other than applying standard proccedures in data preprocessing.
Replication	Not applicable. Our experiments did not aim to uncover any mechanistic or intervention effect. Instead, we benchmarked our proposed methodology against competing methods with different datasets acquired using different technologies.
Randomization	Not applicable. Our experiments did not aim to uncover any mechanistic or intervention effect and hence did not require any controls.
Blinding	Not applicable. Our experiments did not involve human participants and their responses.

Reporting for specific materials, systems and methods

We require information from authors about some types of materials, experimental systems and methods used in many studies. Here, indicate whether each material, system or method listed is relevant to your study. If you are not sure if a list item applies to your research, read the appropriate section before selecting a response.

Materials & experimental systems

n/a	Involved in the study
☒	☐ Antibodies
☒	☐ Eukaryotic cell lines
☒	☐ Palaeontology and archaeology
☐	☒ Animals and other organisms
☒	☐ Clinical data
☒	☐ Dual use research of concern

Methods

n/a	Involved in the study
☒	☐ ChIP-seq
☒	☐ Flow cytometry
☒	☐ MRI-based neuroimaging

Animals and other research organisms

Policy information about studies involving animals; ARRIVE guidelines recommended for reporting animal research, and Sex and Gender in Research

Laboratory animals	N.A.
Wild animals	N.A.
Reporting on sex	N.A
Field-collected samples	N.A
Ethics oversight	N.A

Note that full information on the approval of the study protocol must also be provided in the manuscript.
